# Erratum to “Is p53 Involved in Tissue-Specific Insulin Resistance Formation?”

**DOI:** 10.1155/2017/8036902

**Published:** 2017-09-24

**Authors:** Justyna Strycharz, Jozef Drzewoski, Janusz Szemraj, Agnieszka Sliwinska

**Affiliations:** ^1^Diabetes Student Scientific Society at the Department of Internal Diseases, Diabetology and Clinical Pharmacology, Medical University of Lodz, Lodz, Poland; ^2^Department of Internal Diseases, Diabetology and Clinical Pharmacology, Medical University of Lodz, Lodz, Poland; ^3^Department of Medical Biochemistry, Medical University of Lodz, Lodz, Poland; ^4^Department of Nucleic Acid Biochemistry, Medical University of Lodz, Lodz, Poland

In the article titled “Is p53 Involved in Tissue-Specific Insulin Resistance Formation?” [1], there were a number of language errors, due to publisher error. The corrected version of the article is shown below:

## Abstract

p53 is an extremely versatile molecule, primarily involved in sensing the variety of cellular stresses. Functional p53 utilizes a plethora of mechanisms to protect cells from deleterious repercussions of genotoxic insults, where senescence deserves special attention. While the impressive amount of p53 roles has been perceived solely through the prism of its antioncogenic effect, its presence seems to be vastly connected with metabolic abnormalities underlain by cellular aging, obesity, and inflammation. p53 has been found to regulate multiple biochemical processes such as glycolysis, oxidative phosphorylation, lipolysis, lipogenesis, *β*-oxidation, gluconeogenesis, and glycogen synthesis. Notably, p53-mediated metabolic effects are opposed to insulin action. An accumulating amount of data identifies p53 to be a factor activated upon hyperglycemia or excessive calorie intake, thus contributing to low-grade chronic inflammation and systemic insulin resistance. Prominent signs of its actions have been observed in muscle, liver, pancreas, and adipose tissue, being associated with the attenuation of insulin signalling. p53 is of crucial importance for the regulation of white and brown adipogenesis, simultaneously being a repressor of preadipocyte differentiation. This review provides a profound insight into p53-dependent metabolic actions directed towards promotion of insulin resistance as well as presenting experimental data regarding obesity-induced p53-mediated metabolic abnormalities.

## 1. Introduction

Obesity is now referred to as a global pandemic, and its prevalence is expected to grow in enormously rapid way [1, 2]. As obesity is predominantly driven by environmental factors, it is a subject of extensive epigenetic research [3]. Nevertheless, the genetic aspect of obesity has never been ignored [4–6]. Obesity constitutes an excellent background for a plethora of life-threatening disorders such as type 2 diabetes (T2D), cardiovascular diseases, NAFLD (nonalcoholic fatty liver disease), and many cancers [7–10]. Inevitably, adiposity is tightly linked to metabolic syndrome (MetS). The latter one encompasses a group of metabolically related cardiovascular risk factors, also known to augment the progression of diabetes [11]. The escalating global incidence of T2D is alarming, which is enhanced by the expectation that diabetes will become the 7th leading cause of death in 2030 [12]. Due to such dramatic data, there is a great need to investigate the pathomechanism underlying diabetes, which is always accompanied by numerous vascular complications [13]. p53 is an incredibly versatile molecule mainly involved in sensing cellular stresses, thereby acting as a potent tumour suppressor [14]. An accumulating amount of data suggests that p53 is involved in the pathomechanism of metabolic abnormalities triggered by obesity and hyperglycemia. This review summarizes findings regarding p53's role in the phenomenon of insulin resistance.

## 2. Insulin Signalling Pathway and Insulin Resistance

### 2.1. Insulin Signalling

Insulin is an anabolic hormone involved in the regulation of glucose and lipid homeostasis [15]. Insulin receptor (IR) is a tyrosine kinase receptor, which requires dimerization and binding of insulin molecules for activation. This heterotetrameric receptor is made up of two extracellular *α* subunits and two transmembrane *β* subunits. Autophosphorylation of IR results in the creation of many phosphotyrosine residues, which serve as docking sites for subsequent components of this signalling pathway. PTP-1B (protein-tyrosine phosphatase 1B) utilizes its phosphatase activity to reverse the process of receptor activation, thus being one of several negative regulators of insulin signalling [16]. Multiple phosphotyrosines allow for recruitment and phosphorylation of various substrate proteins, where IRS (insulin-receptor substrate) proteins deserve special attention [17]. Phosphorylated IRSs activate and direct PI3K (phosphatidylinositol-3-kinase) to the plasma membrane. PI3K phosphorylates PIP2 (phosphatidylinositol 4,5-bisphosphate), thereby generating PIP3 (phosphatidylinositol-3,4,5-trisphosphate). PIP3 is the key lipid signalling intermediate undergoing dephosphorylation by two lipid phosphatases, SHIP2 (SH2-containing inositol 5′-phosphatase-2) and PTEN (phosphatase and tensin homolog) [18]. Increased levels of PIP3 activate serine threonine kinase PDK1 (phosphoinositide-dependent protein kinase-1), thus allowing phosphorylation and subsequent activation of PKB/AKT (protein kinase B also known as AKT) and atypical PKC *ζ*/*λ* (protein kinase C) [19, 20]. Both of them increase insulin-induced glucose uptake by triggering translocation of GLUT4 (glucose transporter 4) from intracellular vesicles to the cell membrane. Not only is AKT an antiapoptotic molecule [21], but also its activity results in all metabolic effects mediated by insulin [22]. AKT phosphorylates numerous downstream targets, thereby repressing glycogenolysis, lipolysis, proteolysis, and gluconeogenesis. On the other hand, AKT stimulates lipogenesis, glycogenesis, and protein synthesis. Undoubtedly, insulin is a potent growth factor, triggering cell growth and differentiation [15]. Its mitogenic activity is exerted mainly through stimulation of the mitogen-activated protein kinase (MAPK) cascade. The insulin signalling pathway is depicted in Figure 1.

### 2.2. Insulin Resistance

Insulin action is indispensable for the maintenance of energy balance. It affects a large number of kinases and enzymes during fasting and feeding periods to enable proper functioning of the entire organism. Insulin resistance is the condition characterized by a lowered response of cells to circulating insulin. While the precise molecular mechanism remains to be elucidated, insulin resistance is always manifested by disturbances in insulin signalling, thus leading to its attenuation. There are several molecular pathways that have been suggested to play a great role in this complex metabolic disorder [23]. As obesity is still the major risk factor, the role of free fatty acids is of crucial importance. Physiologically, insulin is secreted from *β* cells upon raised glucose level and acts on tissues, enabling quick storage of energy surplus. Therefore, insulin signalling ultimately leads to translocation of tissue-specific glucose transporters to the cell membrane, causing glucose influx. When calorie intake exceeds demand, energy is primarily deposited in white adipocytes, which are the only cells for safe fat storage [24]. If this state is prolonged, hypertrophy and hyperplasia of adipocytes set in. Hence, the induction of adipose tissue hypoxia leads to low-grade chronic inflammation, which triggers insulin resistance [25]. As insulin cannot further store energy in adipocytes, its raised amount is needed for compensation. Consequently, *β* cells undergo adaptive changes in order to produce and secrete an incredibly large amount of insulin. While insulin sensitivity is decreased, its inhibitory impact on lipolysis is relieved. This results in increased levels of circulating free fatty acids, subsequently taken up by muscles, liver, and pancreas. Thus, all these tissues become affected by lipotoxicity, leading directly to the induction of nonadipose tissue insulin resistance. Then, gluconeogenesis and glycogenolysis are no longer suppressed, triggering glucose output from the liver. Meanwhile, inflammation in adipocytes exacerbates in parallel with their necrosis, due to intensified macrophages infiltration. Hyperinsulinemia leads to *β* cells exhaustion, which makes their function decline. Therefore, the glucose intolerance along with hyperglycemia is established [26]. *β* cell mass reduction up to 60% precedes the first clinical manifestations of type 2 diabetes [27]. Clearly, insulin resistance is closely associated with obesity and is underlain by oxidative stress along with inflammation [26].

## 3. p53 Overview

TP53 gene has been declared the major tumor suppressor gene [28], as the prevailing number of its actions are perceived through the prism of conferring protection to the genome. Consequently, loss of wild type p53 is commonly linked to the phenomenon of tumorigenesis [29], making p53 reactivation a miraculous and promising target in anticancer therapy [30]. This transcription factor is broadly known to be activated in the presence of the plethora of genotoxic insults, like hypoxia, oxidative stress, DNA damage, and oncogene activation, to mention only the most important ones [14]. Depending on the stress severity and the extent of DNA damage, p53 decides whether to save the affected cell or perform the suicidal death to protect it from changing its phenotype to a malignant one. Activated p53 implements an immediate antiproliferative program, yet this is accomplished due to a range of cellular events such as apoptosis, senescence (irreversible cell growth arrest), or cell cycle arrest [14, 31–33]. Induction of cell cycle arrest gives p53 a possibility to perform DNA repair [34], underscoring the relevance of p53 as a mediator of prosurvival and cytoprotective effects. On the other hand, apoptosis and senescence are claimed to be leading ways of p53-mediated tumor suppression, as they are realized when stress causes irreparable and highly hazardous DNA damage. Regardless of the type of implemented actions, p53 always aims to stop the propagation of deleterious mutations. Nevertheless, the exact molecular and mechanistic implications enabling p53 to choose between living or dying still arouse some controversy [35].

TP53 gene product is a broadly known transcriptional activator without being a direct transcriptional repressor [36]. Alternatively, p53 can mediate transrepression by preventing the binding of other transcriptional activators to their target sites [37]. Its nontranscriptional activities involve direct protein-protein interactions with the Bcl2 family of apoptosis-regulatory proteins [38, 39]. Thereby, p53 induces mitochondrial outer membrane permeabilization and cytochrome c release, finally triggering apoptosis.

This major cellular gatekeeper [40] is entangled into the complex gene regulatory network composed of its mediators, effectors, and coregulators [41, 42]. Mediators are sensitizers of stresses, determining p53 actions by marking it with adequate PTMs (posttranslational modifications). In this review, ATM (ataxia teleangiectasia mutated) and AMPK (AMP-activated protein kinase) are of great importance. Effectors constitute a group of more than one hundred genes, which exhibit p53-mediated specific cellular effects. p21 is considered as a master p53 effector, participating in cell cycle arrest and senescence [43, 44]. Its expression level is undoubtedly an indicator of p53 tumour suppressor activity. Moreover, p53 is known to increase expression of proapototic genes such as PUMA (p53-upregulated modulator of apoptosis) [45], NOXA [46], and Bax [47]. While coregulators closely cooperate with p53, they trigger histone modifications as well as changes in chromatin structure [48]. Considering the abundance of p53 target genes and functions, the variety of posttranslational modifications applied to this single protein should not be surprising [49]. Numerous PTMs influence p53 stability and determine its subsequent actions in response to different stimuli, enabling this cellular lifeguard to act quickly and with extreme precision [50].

### 3.1. Regulation of p53 Activity

The “guardian of the genome” is subjected to sophisticated regulation [51]. Namely, its stability and amount are precisely regulated by its closest negative regulator, MDM2 (mouse double minute 2 homolog) [52, 53]. This E3 ubiquitin ligase forms a complex with p53 and determines its fate due to the attachment of ubiquitin molecules. Highly expressed MDM2 conducts p53 polyubiquitination, thereby triggering its nuclear degradation. MDM2-dependent p53 monoubiquitination mediates nuclear exclusion of p53 and precludes its transcriptional activity [54]. Noticeably, p53 ubiquitination is highly dependent on MDM2 activity [54]. When genotoxic stress occurs, MDM2 activity and p53 degradation are repressed. p53 undergoes the process of stabilization and accumulation, followed by tetramerization and binding to target DNA sequences [55]. As p53 induces expression of its closest negative regulator, together they form an autoregulatory feedback loop relevant to the maintenance of balanced cellular conditions. Physiologically, p53 shuttles between two major cellular compartments, the cytoplasm and the nucleus, in a cell-cycle dependent manner [56].

As previously mentioned, AKT is subjected to phosphorylation upon insulin stimulation [57]. Ser473 and Thr308 phosphorylations are demanded for maximal activity of AKT [57]. This molecule has been reported to perform MDM2 Ser166 and Ser186 phosphorylations [58]. Hence, MDM2 entry into the nucleus is promoted [59] and also its self-ubiquitination is limited [60]. Moreover, it decreases p53 transcriptional activity and stimulates MDM2 ubiquitin ligase activity, thereby leading to an increased rate of p53 degradation [61] (Figure 2). When the stressor occurs, p53 aims to block cell growth and survival. Thus, it triggers AKT degradation and transactivates PTEN, a prominent PIP3 phosphatase (Figure 3). Notably, PTEN was demonstrated to physically interact with p53 and promote its acetylation, tetramerization, DNA binding, and transcriptional activity by utilizing phosphate-dependent and phosphate-independent mechanisms [62, 63]. Moreover, PTEN-p53 interaction was potent enough to reverse MDM2-mediated inhibition of p53 [64]. Another research team showed that oroxylin-induced PTEN is capable of reducing the rate of MDM2 transcription and thus favours p53 stabilization [65].

In the case of stressor occurrence ATM kinase undergoes Ser1981 phosphorylation, thereby being released from the inhibitory self-dimer complex [66, 67]. Concomitantly, it influences p53 stability by phosphorylating Ser15 (Ser18 in mice) [68, 69]. In order to preclude p53 degradation, ATM conducts MDM2 Ser395 phosphorylation [70]. Interestingly, ATM was also shown to inhibit MDM2 by affecting its RING domain oligomerization as well as its E3 ligase processivity [71]. In general, ATM activation releases MDM2-mediated inhibitory mechanism, thereby allowing p53 stability (Figure 3). ATM further potentiates p53 activity level by revolving around the suppression of an impressive amount of p53 inhibitors [72]. Prominently, p53 gets immediately deubiquitinylated thanks to USP10 (Ubiquitin Specific Peptidase 10) activity [73]. Moreover, ATM activity elicits cascades of molecular events, going hand-in-hand with the promotion of efficient p53 translation [72]. It is promoted through the binding of MDM2 to p53 mRNA as well as by circumventing MDM2-mediated RPL26 (ribosomal protein L26) degradation [73, 74]. Interestingly, MDM2-p53 mRNA interaction is indispensable for p53 activation triggered upon DNA damage, therefore providing a deeper rationale for the p53-mediated MDM2 induction [75]. This regulatory mechanism adds a new dose of complexity to p53-MDM2 interactions and sheds a new light on the tumour suppressive role of MDM2.

### 3.2. p53 Functions

p53's roles are abundant and extremely diverse (Figure 4) [76]. Different cellular localization and concentration might be factors determining p53 function [77]. p53 is implicated in antioxidant defense [78], suppression, promotion of cellular differentiation [79, 80], and aging [81], among others.

Interestingly, p53 has been declared a “guardian of differentiation” due to its prominent impact on several mesenchymal differentiation programs [79]. Interestingly, p53 plays a dual role in adipogenesis [80]. While it is positively involved in brown adipogenesis, it suppresses white adipocyte differentiation [80].

p53 plays a dual role with respect to oxidative stress phenomenon [77]. Excessive ROS (reactive oxygen species) accumulation induces genomic instability, being a trigger for p53 activation. Consistently, p53 transactivates genes associated with antioxidant defense, such as SESN1 and SESN2 (sestrin 1 and 2, respectively), which reduce the level of ROS and protect cells from the deleterious effects of oxidative stress. Antioxidant genes are subjected to permanent activation, as lack of functional p53 results in increased ROS level, followed by DNA damage [78]. This sequence of events was confirmed in numerous normal and carcinoma cell lines, indicating the fundamental way in which p53 exerts its antioncogenic effect [78]. On the other hand, induction of prooxidant genes is a mechanism used by p53 to induce apoptosis in a ROS-dependent manner [77, 82]. As chronic inflammation underlies the process of tumorigenesis, it is another stimulus for p53 activation [83, 84]. p53 is entangled in a broad crosstalk with inflammatory elements, such as the previously mentioned ROS, cytokines, or NF*κ*B (nuclear factor kappa-light-chain-enhancer of activated B cells) [84]. Although the NF*κ*B-p53 relationship abounds in a variety of mechanisms enabling their mutual repression [85], p53 was suggested to elicit inflammation through the activation of the NF*κ*B pathway [86]. Surprisingly, NF*κ*B might be an indispensable molecule for p53-dependent induction of apoptosis [87].

Moreover, mild oxidative stress intensifies the introduction of single-stranded damage into telomere structures [88]. Damage and shortening of telomeres causes general telomere dysfunctionality, which provokes p53 activation and results in either senescence or apoptosis [89, 90]. Nevertheless, senescence is considered as a major mechanism protecting cells from the effects mediated by this genotoxic stressor [91]. Shortened telomeres are a vivid sign of cellular aging, simultaneously creating a link with p53 signalling. However, the exact role of p53 in aging is still inconclusive and highly debatable, as studies suggest that p53 can function as both its enhancer and repressor [81].

p53 is capable of responding to metabolic stresses. For instance, glucose deprivation is sensed through AMPK (5′AMP-activated protein kinase), which activates p53 and elicits its transcriptional program [92]. Metabolic stressors, which constitute a great threat towards replication fidelity, force p53 to shut down mitogenic signalling expressed by the inhibition of IGF-1/AKT and mTOR (mammalian target of rapamycin kinase) pathways [93]. Depending on the stressor type, p53 was proved to regulate the expression of TSC2 (tuberin), IGF-BP3 (insulin-like growth factor-binding protein 3), and PTEN as well as AMPK [94]. All these molecules negatively affect both the previously mentioned mitogenic pathways in a tissue or cell type specific way. Notably, the stimulation of PTEN and TSC2 expression was especially shown in all insulin-responsive tissues.

As new molecules associated with p53 still enter the arena, knowledge of the intricate nature of the whole regulatory network advances in a very rapid way. Consistently, numerous profound studies still indicate new plausible roles of the guardian of the genome, surpassing expectations of even the most staunch p53 researchers [95].

## 4. p53-Orchestrated Regulation of Metabolism

The versatility of p53 has also dominated metabolism, which gives a new dimension to its antioncogenic actions. Utilizing transcriptional activation as well as other mechanisms, p53 has a great impact on glycolysis, oxidative phosphorylation, nucleotide biosynthesis, and autophagy as well as glutaminolysis or fatty acid oxidation [96]. All in all, p53 has emerged as an essential regulator of metabolic homeostasis [96]. This statement becomes especially prominent in the case of the Warburg effect, which is a pivotal mark of cancer cells. It is manifested by predominant dependence on the glycolytic pathway for ATP synthesis, simultaneously neglecting oxidative phosphorylation. Wild type p53 has a vastly opposing role, expressed by inhibition of glycolysis and promotion of oxidative phosphorylation. As vividly seen, metabolic-oriented actions of p53 are directed towards neutralization of the Warburg effect and the restoration of the physiological way of ATP production. Nevertheless, the increased risk of carcinogenesis in patients suffering from obesity and diabetes gives some clues about a possible link to p53 function [97]. The effects of p53 actions are totally opposed to the effects mediated by insulin (Figure 5); therefore p53 can be perceived as the molecule engaged in creating the state of insulin resistance [98]. Below, examples of p53-orchestrated metabolic regulation are presented.

### 4.1. p53-Dependent Regulation of Glucose Influx, Glycolysis, and Glycogenesis

First, p53 leads to decreased glucose influx by directly repressing expression of two glucose transporters, GLUT1 and GLUT4 [99]. The latter one might also be repressed through an indirect mechanism [100]. Namely, p53 was reported to downregulate PGC-1*α* (peroxisome proliferator-activated receptor gamma coactivator 1*α*), being engaged in the stimulation of GLUT4 expression in muscle tissue [100, 101]. Moreover, p53 indirectly represses GLUT3 expression by influencing another transcription factor, NF*κ*B [102]. The detailed mechanism is focused on the suppression of IKK*α* and IKK*β* kinase activities, being vital components of the NF*κ*B signalling cascade.

Additionally, p53 affects the first component of insulin signalling. Namely, insulin receptor (IR) promoter has been shown to undergo repression mediated by p53 and accompanied by two other factors, C/EBP (CCAAT-enhancer binding proteins) and Sp1 (specificity protein 1) [103].

p53-stimulated repression of glycolysis is executed in several ways. First, it performs inhibition of hexokinases HK1 and HK2 and phosphoglycerate mutase (PGAM), which catalyze the first and eighth glycolysis steps [104, 105]. The latter one is not subjected to p53 transcriptional repression, yet its protein stability is restricted [105]. Hexokinases' upregulation is one of the most significant signs of the cancerous phenotype [106], simultaneously being the most crucial enzymes for glucose metabolism. p53 represses hexokinases and phosphoglycerate mutase by inducing the expression of miR-34a [104]. Nevertheless, these enzymes can be likewise subjected to opposite regulation, thus creating evidence for p53-stimulated positive regulation of glycolysis [107, 108]. The above aspect highlights the relevance and complexity of p53 actions. Importantly, the p53-miR34 signalling pathway also contributes to the repression of GPI (glucose-6-phosphate isomerase), which catalyzes the second glycolytic reaction. Moreover, p53 overexpression itself has been reported to correlate with the decrease of another glycolytic enzyme, aldolase C (ALDC) [104].

TIGAR (TP53-induced glycolysis and apoptosis regulator) is a downstream p53 effector, which acts as a suppressor of phosphofructokinase 1 (PFK) [109]. PFK catalyzes the third step of the glycolytic pathway, simultaneously being the rate limiting enzyme undergoing allosteric regulation by fructose-2,6-bisphosphatase. TIGAR acts as fructose-2,6-bisphosphatase, which causes the accumulation of fructose-6-phosphate followed by its isomerization to glucose-6-phosphate. Not only is TIGAR claimed to be a glycolysis inhibitor, but also the pentose phosphate pathway (PPP) enhancer is. Additionally, it provides protection against ROS-dependent damage by the production of NADPH needed for the formation of reduced glutathione [109]. TIGAR has been shown to activate HK2 activity, which can be perceived as a plausible mechanism enabling complex direction of glycolytic intermediates to the pentose phosphate pathway, thus causing even more profound repression of aerobic glucose oxidation [110].

RRAD (Ras-related associated with diabetes) was revealed to be overexpressed in some T2D patients [111]. Furthermore, it is likewise responsible for the reduction of glucose uptake in response to insulin stimulation in muscle and adipose tissue [112]. Recently, it was found to be a p53 target gene engaged in the repression of glycolysis under hypoxic conditions [113, 114]. The exact mechanism is centered on preventing the translocation of GLUT1 to the cell membrane. Although the study was conducted on lung cancer tissue, RRAD might be also subjected to p53 regulation in insulin-responsive tissues.

p53 was connected with restrained rate of glycogen synthesis in insulin-resistant subjects [115]. Study data provided by Fang et al. indicated that p63, which is a member of the p53 family, is directly targeted by mir-20a-5p. Reduced amounts of this miRNA led to increased expression of p63, followed by its physical interaction with p53 and thus leading to an increase of p53 and PTEN. Restriction of glycogenesis was proved to be conducted through PTEN-mediated AKT/GSK pathway inhibition.

### 4.2. p53-Mediated Enhancement of Oxidative Phosphorylation

Altogether, p53 is perceived as a negative glycolysis regulator due to direct as well indirect repression of essential glycolytic enzymes. p53 also performs glycolytic pathway limitation by profoundly promoting oxidative phosphorylation for ATP synthesis. The stimulation of glutaminolysis and fatty acid oxidation (FAO) as well as direct induction of genes influencing mitochondrial respiratory chain complexes vastly ameliorates this metabolic switch. Furthermore, such metabolic control stays in agreement with p53 tumour suppressive responsibility and presents a totally opposite effect in comparison to insulin signalling (Figure 5).

In physiological conditions, pyruvate, an end product of glycolysis, is converted to acetyl-CoA. The latter serves as the entry molecule in the tricarboxylic acid cycle (TCA). p53 increases the rate of acetyl-CoA formation due to direct regulation of genes affecting pyruvate dehydrogenase (PDH), the first enzymatic component of the pyruvate dehydrogenase complex. p53 decreases the expression of PDK1 and PDK2 (pyruvate dehydrogenase kinase 1 and pyruvate dehydrogenase kinase 2, respectively), known to conduct repressive phosphorylation of PDH [104, 116]. In contrast, p53 was shown to increase the expression of PARK2 (parkin), which positively regulates PDH activity as well as strengthening cellular antioxidant defense [117]. Moreover, p53-mediated repression of lactate dehydrogenase A (LDHA) protects pyruvate from conversion to lactate, thus further promoting the formation of acetyl-CoA [104].

p53 affects the tricarboxylic acid cycle by directly inducing the expression of mitochondrial GLS2 (glutaminase 2), which is commonly known to conduct the process of glutamine hydrolysis. This results in the formation of glutamate and a consequent increase in the *α*-ketoglutarate level, a component of TCA. Consequently, p53 intensifies mitochondrial respiration and the subsequent ATP production rate [118].

A direct impact of p53 on oxidative phosphorylation is executed through the enhancement of SCO2 (synthesis of cytochrome c oxidase) expression level. The latter one regulates the COX (cytochrome c oxidase) complex, a pivotal component of oxygen utilization [119]. AIF (apoptosis-inducing factor) is a mitochondrial flavoprotein presumed to positively affect the mitochondrial respiratory complex I. Crucially, p53 performs constitutive activation of AIF expression, thereby allowing efficient oxidative phosphorylation [120, 121].

### 4.3. Impact of p53 on the Regulation of Gluconeogenesis

As insulin exerts a repressive effect on gluconeogenesis, it is worthwhile to accurately establish the role of p53 in gluconeogenic regulation. Firstly, the liver p53 level seems to be dynamically regulated by nutrient availability, where activation of p53 and its target genes occurs in the case of food withdrawal through the AMPK-mediated mechanism [122]. Another point is that p53 was implicated to be a central node governing the regulation of fasting in critical metabolic tissues, such as liver, muscles, and adipose tissue [123]. Interestingly, the absence of functional hepatic p53 results in defective gluconeogenesis thus promoting hypoglycemia [122]. However, study data implicates both p53-induced suppressive [124, 125] and stimulatory [126–128] effects in de novo glucose production. The most recent findings suggest that p53 directly activates sirtuin 6 (SIRT6), which in turn facilitates export of FoxO1 (forkhead box protein O1) to the cytoplasm [124]. Consequently, FoxO1 is not capable of enhancing the expression of two vast gluconeogenic executors, PCK1 (phosphoenolpyruvate carboxykinase 1) and G6PC (glucose 6-phosphatase). Nevertheless, the accumulating amount of contradictory data reflects the need for further confirmation of the exact role of p53 in the regulation of this particular metabolic pathway.

### 4.4. Implications of p53 in the General Glucose Homeostasis

As seen above, a large body of evidence indicates the vital influence of p53 on glucose metabolism. Besides biochemical implications, p53 signalling seems to underlie general physiological glucose homeostasis [129, 130]. Its impact has been observed in patients with A-T (ataxia teleangiectasia), who are highly susceptible to insulin resistance state, followed by the development of T2D [131]. ATM protein is the trigger for A-T disease, simultaneously being considered as a molecular target in metabolic associated disorders [132, 133]. It also acts as an upstream mediator of p53 function [134]. As ATM kinase performs p53 activation through Ser18 phosphorylation, the mouse model with p53S18A was used to check the influence of the ATM-p53 signalling pathway on the physiological regulation of insulin sensitivity [129]. This molecular defect turned out to be responsible for metabolic stress, accompanied by glucose intolerance and subsequent insulin resistance. Insulin signalling was markedly reduced and associated with increased ROS level, as this metabolic imbalance was reversed by antioxidant treatment [129]. Additionally, it was revealed that a higher dose of p53 transactivation domain contributes to intensified protection against any disturbances concerning glucose homeostasis [130]. In this study a specific mouse model with p53 Ser18 defect was utilized, enabling observation of effects associated with the loss of p53-dependent activation of hzf (hematopoietic zinc finger protein) in white adipose tissue [135]. Notably, hzf is an important adipogenesis regulator. Mice exhibited adipose tissue specific insulin resistance as well as an increased level of TNF-*α* along with a notable decrease in adiponectin level. The above results confirm the significance of ATM-p53 signalling in the maintenance of glucose homeostasis. Additionally, it exceptionally underscores the value of adipose tissue functionality, which if disturbed leads directly to insulin resistance and inflammation.

On the other hand, p53 knockout mice did not exhibit any differences in insulin, glucose, and pyruvate tolerance tests in comparison to wild type mice [136]. Insulin-stimulated glucose uptake was not changed in adipocytes with siRNA-suppressed p53 expression. Totally different findings originated from studies performed on identically treated hepatocytes. Although glucose uptake turned out to be diminished, there were no changes in the expression of GLUT1 and GLUT2 in comparison to control hepatocytes. Similarly, the level of phosphorylated AKT was found to be equal in both types of cells, leaving the exact p53-mediated mechanism remaining to be elucidated. Nevertheless, p53 itself was not indispensable for general maintenance of glucose homeostasis [136]. Intriguingly, a lowered serum level of TP53 gene product was reported in diabetic subjects as well as patients with impaired glucose tolerance [137].

### 4.5. p53-Induced Regulation of Fatty Acid Metabolism

p53 has been observed to play a key role in both lipid catabolism and fatty acid *β*-oxidation [138]. It contributes to the first metabolic pathway by directly stimulating molecules engaged in the release of fatty acids from lipids. Namely, it activates proteins such as cytochromes P450-4F2 and 4F3, carnitine O-octanoyltransferase (CROT), and two types of carnitine palmityltransferase, CPT1A and CPT1C [138–141]. Carnitine acetyltransferases are responsible for conjugating fatty acids to carnitine, which enables their transport to the mitochondrial matrix [141]. Cytochromes P450-4F subject long fatty chain acids to hydroxylation, leading to *β*-oxidation [139].

Starvation-induced lipolysis was reported to be governed by p53 through stimulation of its downstream target Ddit4 (DNA Damage Inducible Transcript 4) in adipocytes [123]. The relationship between p53 and lipolysis was likewise associated with chronic pressure overload and DNA damage in obese adipocytes [142, 143].

Considering p53-mediated fatty acid oxidation regulation, it must be stressed that all of them undergo activation in the glucose deprivation state. LPIN1 (lipin 1) and GAMT (guanidinoacetate N-methyltransferase) expression levels are subjected to p53-dependent increase [144, 145]. LPIN1 exhibits cooperation with two transcriptional regulators, PPAR*α* (peroxisome proliferator-activated receptor alpha) as well as PGC-1*α* [146]. Their combined cellular effect is associated with the inhibition of fatty acid synthesis and the enhancement of fatty acid oxidation. As mentioned before, p53 has been revealed to directly repress and interact with PGC-1*α*, one of the master metabolic gene expression coactivators [101]. Additionally, PGC-1*α* is a p53 regulator, forcing it to elicit transcriptional activation of genes involved in the metabolic regulation and cell cycle arrest [147]. GAMT expression induction results in a vast increase of FAO rate by a mechanism involving stimulation of creatine synthesis [145].

PANK1 (pantothenate kinase-1) influences the level of intracellular CoA (coenzyme A) by catalyzing the rate limiting step in the CoA synthesis pathway [126]. It has been reported to be a direct positively regulated p53 transcriptional target gene. Mice lacking functional PANK1 and subjected to starvation demonstrated defective *β*-oxidation and gluconeogenesis. Therefore, p53 was implicated to be an enhancer of both of these processes.

Intriguingly, FAO induction is also mediated by the RP-MDM2-p53 metabolic response pathway. RP (ribosomal protein) becomes activated upon suppression of ribosomal biogenesis occurring during metabolic stress. As RP binds MDM2, it contributes to subsequent p53 induction [148]. Finally, MCD (malonyl-coenzyme A decarboxylase), critically involved in the process of FAO, undergoes p53-dependent upregulation. The RP-MDM2-p53 signalling pathway has a promoting impact on mitochondrial fatty acid uptake, as MCD regulates malonyl-CoA turnover and subsequent CPT1*α* activation.

Actions of the master tumour suppressor are also directed towards inhibition of lipid anabolism. Namely, G6PD (glucose-6-phosphate dehydrogenase) is markedly repressed upon p53 stimulation, which leads to lowered efficiency of the pentose phosphate pathway (PPP) and consequently to reduced NADPH levels. These events create a cellular signal, which decreases lipogenesis [149].

SREBP1c (sterol regulatory element-binding protein 1c), which belongs to the family of transcription factors regulating adipogenesis and lipogenesis [150, 151], is subjected to p53-dependent downregulation [152]. SREBP1c stimulates the expression of FASN (fatty acid synthase) as well as ACLY (ATP citrate lyase), which were likewise revealed to undergo p53-mediated repression [138, 152].

Besides direct regulation of metabolic enzymes, hypothalamic SIRT1/p53 pathway was implicated in the response towards ghrelin, the hormone regulating food intake [153]. However, mice lacking functional p53 were not characterized by impaired ghrelin-dependent stimulation of growth hormone secretion. Moreover, p53-mediated regulation of adipogenic and lipogenic genes was found to be indispensable for ghrelin-induced storage of lipids in adipose tissue and liver [154].

## 5. Tissue-Specific p53-Mediated Changes Triggered by Obesity and Diabetic Conditions

Another piece of evidence linking p53 with glucose metabolism is associated with its response towards glucose oscillations as well as hyperglycemic stimuli. It has been established that hyperglycemia activates p53 and its downstream mediators in myocyte cells, leading to their apoptosis [155]. Additionally, many studies have shown p53 activation upon high-fat and high-calorie diet treatment. Discovery of the new triggers for p53 induction encouraged the scientific community to investigate p53's role in multiple tissues.

### 5.1. p53 Role in the Pancreas

The relationship between p53-dependent apoptosis, hyperglycemia and mitochondrial dysfunction was widely investigated in pancreatic RINm5F cells [156–158]. First, it is clear that hyperglycemia causes oxidative stress, contributing to pancreatic *β* cell apoptosis [159]. The suggested mechanism is thought to be based on mitochondrial p53 mobilization, leading further to decreased mitochondrial membrane potential, followed by cytochrome c release and fragmentation of nuclear DNA [156]. Another study implied a plausible link between p53 Ser392 phosphorylation and p38 MAPK activation in hyperglycemia-stimulated *β* cells mass decrease [157, 160]. Inhibition of p38 MAPK activity coincided with the blockage of p53 translocation and decreased rate of apoptosis. In other words, p53 Ser392 phosphorylation was proved to be stimulated upon hyperglycemia and favors p53 mitochondrial translocation. In the last similar study, the MDM2-p53 regulatory status as well ATM and AKT regulation was examined [158]. AKT was found to undergo hyperglycemia-dependent phosphorylation and phosphorylated MDM2 at Ser166 [57, 59–61, 158]. Nevertheless, MDM2 mRNA expression and protein concentration were vastly decreased. High glucose seemed to support the formation of an MDM2-p53 inhibitory complex in the cytoplasm, without any influence on its nuclear formation. ATM nuclear phosphorylation was reported to coincide with p53 Ser15 phosphorylation, indicating p53 activation [68, 69]. Despite the formation of a MDM2-p53 complex, p53 was stabilized and its ubiquitination rate was limited. In a recent review, the authors suggest many plausible mechanisms adversely affecting the MDM2-p53 autoinhibitory feedback loop [161]. It seems that MDM2 undergoes activation upon high glucose stimulus, but its ubiquitin ligase activity is disturbed. This might happen due to hyperglycemia-induced oxidative stress and consequent multiple phosphorylation events. Special emphasis is placed on the role of ATM kinase, which could impair MDM2 ubiquitin ligase activity by the phosphorylation of some residues in RING-finger and acid central domains. Moreover, p53 polyubiquitination can be reduced due to ROS-dependent limitation of cellular ATP levels. According to the authors, hyperglycemia supports the formation of p53 PTMs such as phosphorylation, poly ADP-ribosylation, and O-N-acetylglucosaminylation, which probably contribute to its stabilization and subsequent translocation to mitochondria [162]. All these findings are schematically depicted in Figure 6.

*β* cell apoptosis was found to be associated with AGE-RAGE pathway [163]. Not only can AGEs (advanced glycation end products) originate from food, but they can also be formed in the process of nonenzymatic glycation of proteins. RAGE (receptor for advanced glycation end products) is a receptor known to bind glycated proteins. An elevated level of AGEs is one of the most vivid signs of chronic hyperglycemia, supporting pathogenesis of diabetic complications [164]. p53 was also found critical for apoptosis of *β* cells in a glycation-serum-induced mechanism [165]. Studies performed on INS-1 cells and primary rat islets indicated that transcriptional activity of p53 becomes substantially increased in the presence of glycation serum (GS) and contributes to cell apoptosis. Intriguingly, *β* cell demise was avoided upon p53 inhibition, which underlined the relationship between this major tumour suppressor and the AGE-RAGE pathway.

In a study investigating mitochondrial functionality along with GSIS (glucose induced insulin secretion), MDM2 and p53 expression levels in *β* cells originating from diabetic models were found vastly upregulated [166]. Lack of p53 inhibition provoked repression of pyruvate carboxylase (PC), a mitochondrial enzyme engaged in the production of oxaloacetate and NADPH. Downregulation of these TCA cycle intermediates set the scene for impaired oxygen consumption, ultimately leading to dysfunctional mitochondrial metabolism and defective GSIS. Given the obtained results, the conjecture of differential p53 roles depending on the stage of T2D was made. While a slight increase in p53 level could repress GSIS, the progression of diabetes could further stimulate p53 upregulation and evoke *β* cell apoptosis, thereby causing hyperglycaemia.

Moreover, glucolipotoxicity was shown to be a contributor to increased levels of cytoplasmic p53 due to both oxidative and ER stress [167]. The suppressive interaction between p53 and Parkin was revealed to be a culprit for impaired mitophagy in concert with the disturbance of the electron system transport and a consequent cellular ATP deficit. This sequence of events eventually resulted in glucose intolerance and impaired insulin secretion in animal models of both types of diabetes. While the MDM2-p53 relationship still appears intriguing, another ubiquitin ligase, ARF-BP1, was reported to be critical for p53 activity in the context of age-related pancreatic *β* cell viability and homeostasis. In support of this statement, activated p53 was proved to elicit acute diabetic symptoms concurrently with shortening the life span* of arf-bp1* mutant mice [168].

According to Tavana et al., NHEJ-p53R172P mutant mice, which demonstrated a combination of deficient p53 and nonhomologous end-joining (NHEJ), exhibited severe diabetes and consequentially early death [169]. Accumulation of DNA damage induced the increase of p53 and p21, which led to cellular senescence along with the reduction of *β* cell proliferation and overall depletion of pancreatic islet mass. Other studies suggested that independently of the stimulus causing *β* cell metabolic stress, the formation of DNA double-strand breaks along with consequent p53 induction constitutes the main mechanism leading to *β* cell apoptosis [170]. Revolving around mechanistic details of p53 abundance, mir-200 dosage was delineated as a p53-amplifying factor promoting islet apoptosis in diabetic mice [171]. Another study provided an insight into the role of Δ40p53, the 44 kD p53 isoform with a truncated transactivation domain in the context of *β* cell biology [172]. Δ40p53 is engaged in setting the balance between all p53 isoforms. Its overexpression endows cells with highly stabilised p53, thereby instigating accelerated aging and attenuated cell proliferation, as well as promoting some abnormalities in terms of insulin/IGF-1 signalling [172]. Although Δ40p53 has no transcriptional activity, it does impact full-length p53 activity due to its ability to form tetramers. Study results proved that Δ40p53 affected *β* cell proliferation, leading further to *β* cell mass decrease. Glucose intolerance along with hypoinsulinemia was established in 3-month old mice, and the effect progressed with age ultimately leading to overt diabetes and death. An increased level of p21 gene expression was also revealed. Intriguingly, in another study, stress-activated p21 was proved to evoke *β* cell mass decrease through stimulation of intrinsic apoptotic pathway [173]. By contrast, p21 overexpression was found to be critical for *β* cell recovery through the suppression of *β* cell duplication after streptozotocin treatment [174]. Consistently, deficiency or genetic ablation of p21 deepened the effect of glucotoxicity on *β* cell apoptosis, simultaneously promoting diet-induced diabetes [175]. The use of nutlin-3a, the inhibitor of the interaction between p53 and MDM2, stimulated p21 expression, thus preventing ER-stress-mediated effects of diabetic milieu conditions and protecting *β* cells from apoptosis. Similarly, nutlin-3, as an activator of p53, was indicated to ameliorate streptozotocin-induced DM and proposed to provide antidiabetic effects [176], consequently raising some questions about the role of the p53/MDM2 relationship.

Moreover, p53 was found to be entangled in the causal relationship between *β* cell demise and lipotoxicity through free fatty acid- (FFA-) dependent reduced activation of AKT and a simultaneous increase in p53 levels [177]. Chronic FFA exposure was shown to stimulate p53 and consequential transcriptional activation of mir-34a in *β* cells, which elucidated an alternative mechanism of *β* cell apoptosis [178]. In another study, stearic acid contributed to the activation of PERK (protein kinase-like endoplasmic reticulum kinase), which stimulated p53 for induction of mir-34a-5p in islets of diabetic mice and in vitro studies [179]. Finally, lipotoxicity was evoked through consequential reduction of BCL-2 (B cell CLL/lymphoma 2) and BCL-W (BCL-2-like 2), direct targets of mir-34a-5p. It resulted in not only the potentiation of the susceptibility of *β* cells to apoptosis, but also in the impairment of insulin secretion.

TCF7L2 (T-cell factor 7-like 2) is a transcription factor, which has attracted a great deal of interest recently due to its causative impact on *β* cell pathophysiology as well as its association with T2D [180]. Its contribution towards disturbed insulin secretion, incretin effect, and consequential lowered *β* cell survival was enriched by the molecular link with the major tumor suppressor gene. Namely, TCF7L2 was identified to employ the p53-p53INP1 molecular pathway to negatively modulate the fate of islet *β* cells [180].

Although the link between type 1 diabetes and p53 is beyond the scope of this review, potentiated activity of p53 was likewise unequivocally demonstrated in the context of this type of *β* cell dysfunction [181]. The whole mechanism was underlain by the presence of ROS and inflammatory cytokines, which induced the activation of JNK and SAPK. GAD65 (glutamic acid decarboxylase 65 KDa isoform), which is a remarkably significant autoantigen underpinning the pathomechanism of diabetes type 1, was reported to be subject to p53 regulation [182].

### 5.2. p53 Role in the Endothelium and Muscle Tissue

Taking into account the consequences of diabetes and obesity, the role of endothelial dysfunction should not remain undervalued. High-glucose incubation of endothelial cells evoked the reduction of SIRT1 in concert with higher acetylation and activation of p53, which provoked the senescence and dysfunction of endothelial cells [183]. The same results were obtained in vivo [184]. Endothelial “metabolic memory” formation in the presence of high glucose oscillations is also being connected with p53. Here, the crucial role for the p53-MDM2 inhibitory feedback loop as well as prolonged activation of its target genes after stressor removal is specifically underlined [185]. Mitochondrial localization of p53 is supposed to underlie metabolic memory phenomenon, leading further to organelle dysfunction and oxidative stress accompanied by mtDNA damage [155]. Additionally, p53's remarkably negative impact has been noticed in endothelial progenitor cells (EPCs) [186]. Both EPCs cultured in diabetic milieu and EPCs from diabetic donors exhibited accelerated senescence-like changes. This effect coexisted with the activation of the AKT/p53/p21 signalling pathway, pointing out the major role of p53 in the impairment of diabetic neovascularization. On the other hand, a diet-induced obesity mouse model enabled the demonstration of endothelial p53 as an important inactivator of endothelial nitric oxide synthase (eNOS) [187]. Further studies showed an increase in p53-dependent PTEN transactivation, consequently affecting the Akt-eNOS pathway. Subsequently, it decreased the expression of PGC-1*α* in skeletal muscle, followed by a markedly reduced level of mitochondrial biogenesis and energy consumption. Additionally, deletion of endothelial p53 was manifested by increased muscle glucose uptake, due to a lack of p53-GLUT1 inhibitory regulation. The upregulation of endothelial p53 was claimed to be a causative agent of all observed metabolic abnormalities, including intensification of insulin resistance as well as fat accumulation. Promisingly, exercise promoted downregulation of p53 and TIGAR in skeletal muscle of Goto-Kakizaki (GK) rats without changes in SCO2 expression [188]. Therefore, exercise implementation constitutes a weapon against the hazardous activity of p53, leading to lowered levels of oxidative stress along with enhanced levels of mitochondrial DNA content.

### 5.3. p53 Role in the Formation of Insulin Resistance in the Liver

NAFLD (nonalcoholic fatty liver disease) is the general term defining liver abnormalities such as hepatic steatosis and nonalcoholic steatohepatitis (NASH), which can lead to fibrosis, cirrhosis, and hepatocellular carcinoma [189]. NAFLD is associated with insulin resistance due to hepatic triglyceride accumulation, connected with raised levels of circulating free fatty acids. Both of these factors further exacerbate the inflammation state, ultimately forming a vicious cycle [190]. Upregulated p53 was confirmed to induce cytotoxicity, exacerbating liver injury in various mouse models of NAFLD [191]. Another study investigated the impact of p53 on apoptosis, which is a basic mechanism favouring hepatocyte elimination in NAFLD [192]. The amount of p53 was dependent on the severity of liver steatosis and thus linked to inflammation. While p53 upregulation was parallel with downregulation of antiapoptotic Bcl-2, proapoptotic Bax expression was not changed. A NASH mouse model exhibited p53 activation along with changes of its downstream effectors [193]. The induction of p21, suppression of antiapoptotic Bcl-XL, and formation of t-Bid confirmed the role of p53 in the stimulation of intrinsic and extrinsic mitochondrial apoptosis pathways. The same nutritional NASH model revealed that the induction of p53 enhances p66Shc signalling, ROS accumulation, and hepatocyte apoptosis, thereby promoting the progression of steatohepatitis [194]. The expression changes obtained in this mouse model were confirmed by evaluation of liver samples from patients characterised by different severities of NAFLD. Other findings connected NAFLD pathology with the p53/miRNA34a pathway, known to repress SIRT1 (sirtuin1) expression [195]. A selective p53 transcriptional activity inhibitor called pifithrin-*α* (PFT) was administered to a high-fat diet-induced NAFLD mouse model. As the p53-mediated inhibitory impact was relieved, the SIRT1/PGC1*α*/PPAR*α* and SIRT1/LKB1/AMPK pathways were activated. Consequently, malonyl-CoA decarboxylase (MLYCD) underwent activation, leading to the reduction of malonyl-CoA concentration. As mentioned above, this step is always followed by the enhancement of CPT1 activity, thereby inducing *β*-oxidation of fatty acids. Notably, the level of hepatic fat accumulation was diminished, which was manifested by the lessening of steatosis, oxidative stress and apoptosis. Intriguingly, PFT served as a factor lowering overall fat accumulation upon high-fat diet treatment [195]. Increased levels of p53 in hepatic cells were reported in a streptozotocin-induced animal model of diabetes [196]. All of the above-mentioned p53-based mechanisms leading to hepatocytes' apoptosis in the context of NAFLD are presented in Figure 7.

p53 level was upregulated by the treatment of hepatocytes with saturated fatty acids (SFA) along with its inhibition resulting in the prevention of SFA-induced cell death [197]. Once again, the antagonism between p53 and insulin activities was strongly highlighted, as insulin treatment antagonized the deleterious effects of p53. Moreover, as mentioned above, p53 overexpression in livers of diabetic or NAFLD patients could be caused by the downregulation of mir-20a-5p, consequent suppression of glycogen synthesis and the introduction of hepatic insulin resistance [115].

The previously observed connection between hepatic fibrosis and p53 accumulation in patients was investigated on, among others, hepatocyte-specific Mdm2-knockout mice [198]. Relief from MDM2 suppression prompted p53 to elicit spontaneous hepatic fibrosis. This liver dysfunction did not occur when p53 was subjected to concomitant deletion. p53 indirectly promoted the synthesis of CTGF (connective tissue growth factor), which is a molecule notorious for its relevant participation in fibrosis pathophysiology, via downregulation of the miR-17-92 cluster gene [198].

Surprisingly, the effect of p53 activity measured in the context of glucose homeostasis in insulin-resistant diabetic mice turned out to be highly beneficial and was described as an insulin-like antidiabetic effect [199]. Overexpressed p53 improved a plethora of biochemical indicators, such as levels of glucose and serum triglycerides. Furthermore, glycogenesis genes were subjected to substantial upregulation concomitantly with downregulation of gluconeogenesis-associated ones. Positive changes in hepatic and pancreatic gene expression levels were reported for insulin receptor precursor, PPAR-*γ*, GLUT2, and GK (glycerol kinase). All in all, these results are in strong opposition to previous findings, thus demonstrating miraculous p53-stimulated effect on glucose homeostasis with a special focus on the amelioration of hepatic insulin sensitivity.

### 5.4. p53 Role in the Formation of Insulin Resistance in Adipose Tissue

The very first implication for p53 elevation in classic insulin-responsive tissues was shown in adipocytes of genetically obese (*ob*/*ob*) mice [152]. The study aimed at evaluating expression of p53 and its target genes (p21, MDM2-2, Bax, and IGFBP-3) during fasting and refeeding periods. Surprisingly, the expression of all enumerated molecules was proved to be increased upon refeeding stimuli. In this study, SREBP-1c was revealed to be downregulated by p53, highlighting the role of p53 as an essential lipogenesis repressor. The substantially elevated level of p53 was vividly linked with obesity-induced insulin resistance, creating a growing number of questions associated with the exact nature of its adipose tissue existence.

The groundbreaking study conducted by Minamino et al. established the outstandingly important role of adipose tissue p53 upregulation in augmenting insulin resistance [127]. Most of all, white adipose tissue originating from high-fat-diet treated Ay mice exhibited p53 and p21 expression increase. This observation suggested the induction of a senescence-like phenotype. Moreover, the excessive levels of TNF-*α* (tumour necrosis factor) and CCL2 (chemokine C-C motif ligand 2) were found to occur due to the senescence of both macrophages and adipocytes. The comparison of the results obtained from two mice strains, Ay* Trp53*^+/−^ and Ay* Trp53*^+/+^, provided ultimate evidence for p53 as a factor strongly exacerbating insulin resistance and glucose intolerance as well as inducing increased insulin plasma levels. Additionally, it has established p53's role in enhancing expression of hepatic gluconeogenic enzymes. p53 was likewise indicated to participate in the formation of telomere-dependent insulin resistance. On the other hand, adipo-p53-deficient mice exhibited proper insulin signalling due to the restoration of insulin-dependent AKT phosphorylation. Results demonstrated that the presence of ROS leads to p53 activation, followed by NF*κ*B-dependent induction of proinflammatory cytokines. Finally, studies conducted on human adipose tissue removed during abdominal surgery from diabetic and nondiabetic patients confirmed the increased level of p53 protein and CDKN1A mRNA expression. Significantly, inflammatory cytokines were elevated in adipose tissue originating from diabetic patients. All of the above results point to a complex role of p53 in the pathophysiology of insulin resistance, connecting it with inflammation, telomere shortening, and senescence of adipose tissue. In this study, the prominent role of cellular aging is highlighted, shedding a new light on age-related obesity and insulin resistance. Further studies conducted by this research group found evidence suggesting that Sema3E (semaphorin 3E) and its receptor, plexinD1, may play a role in creating adipose tissue inflammation upon p53 activation in a diet-induced obesity mouse model [200]. Sema3E is a secreted protein, transcriptionally activated by p53. It was revealed to act as a chemoattractant, thereby directly triggering visceral fat inflammation along with all metabolic abnormalities exacerbating insulin resistance. Thus, Sema3E is suggested to be another potential therapeutic target in the fight against insulin resistance. Findings originating from these highly relevant studies are visualized in Figure 8.

An intriguing relationship between p53 and hyperglycemia was observed in studies investigating the influence of AGEs on senescent preadipocytes [201]. RAGE-AGE axis was demonstrated to restore adipogenic function of senescent preadipocytes, whose adipogenic potential had been repressed by age-related p53 activity. Glycated proteins stimulated RAGE to suppress p53 expression and modify its functionality due to direct protein-protein interactions. Altogether, it seems that p53-stimulated preadipocyte senescence is a mechanism providing protection against excessive age-related adiposity, which might be reversed by AGE-RAGE axis.

Aside from hyperglycemia, hyperinsulinemia was also recognized as a factor strongly potentiating p53 activity [202]. The study conducted on 3T3-L1 adipocytes proved that increased p53 expression in concert with a decreased level of phospho-MDM2 disturbed insulin signalling and glucose uptake, thus promoting hyperinsulinemia-induced insulin resistance. Prominently, p53 was subjected to time-dependent enhancement without signs of cytotoxicity.

Adipose tissue p53 elevation has been reported to associate with chronic pressure overload [142]. The pathomechanism was mediated by sympathetic nervous stimulation, subsequently resulting in lipolysis promotion. Notably increased levels of free fatty acids triggered adipocytic p53 overexpression and promoted upregulation of proinflammatory cytokines in a NF*κ*B-dependent manner. All of the above-mentioned events elicited adipose tissue inflammation, followed by insulin resistance and successive hyperinsulinemia. Ultimately, p53 accumulation led to heart failure, simultaneously forming a metabolic vicious cycle. The detailed mechanism of p53-induced inflammation upon pressure overload concerning endothelial and bone morrow cells has been elucidated, confirming its role in cardiac dysfunction [203]. Diabetic cardiomyopathy was also demonstrated to be underlain by diabetes-induced ROS-mediated increased levels of p53 and SCO2. Changes in gene expression extensively stimulated mitochondrial oxygen consumption for undue generation of ROS, thereby supporting lipid accumulation and cardiac dysfunction [204]. Cardiomyopathy of diabetic subjects was accompanied by raised levels of p53 and p21 and decreased levels of mir-181a and mir-30c, which were validated to directly target p53 [205].

The results provided by Vergoni et al. strongly suggested that ROS-induced DNA oxidation occurring in the early onset of obesity was a trigger for p53 activation in obese adipocytes [143]. Studies conducted on 3T3-L1 cells as well as mouse models implied that raised levels of p53 and p21 were simply a response to DNA damage. These molecular shifts were followed by a classic cascade of events leading to insulin resistance such as adipose tissue inflammation, increased macrophage chemotaxis, impaired insulin downstream signalling along with insulin-induced glucose uptake, and increased lipolysis in affected adipocytes. Conceivably, adipocytes were not apoptotic due to the protective effect of raised p21.

Bogazzi and colleagues, who examined male C57BL/6J × CBA mice, proved that adipose p53 and growth hormone (GH) pathways converge, which evoked the insulin-resistant phenotype upon high-fat diet treatment [206]. Blockage of GH through the p38-MAPK pathway was potent enough for normalization of p53 levels in adipose depots, suggesting that GH action is indispensable for p53-triggered metabolic repercussions.

In contrast, a study conducted by Ortega et al. provided totally unexpected and contradictory results [207]. The study was focused on the evaluation of adipose tissue p53 expression depending on inflammation and insulin resistance. Impressively, the results were obtained from subjects characterized by a distinct degree of obesity and severity of insulin resistance as well as for several in vivo and in vitro models. The authors postulated that the upregulation of p53 is solely associated with factors triggering inflammation, such as senescence-connected shortening of telomeres or progressive obesity. Surprisingly, the expression of adipose tissue p53 was inversely correlated with insulin resistance and hyperglycemia. Special attention was also focused on the level of p53 during adipogenesis, which was found to be increasingly repressed. Herein, the causal connection between inflammation and p53 expression was once again underlined, as mature adipocytes produce a significant amount of anti-inflammatory adipokines. High glucose level was another factor that intensified p53 downregulation in preadipocytes. Taken together, p53 was subjected to dual and opposed regulation. While inflammation stimulated its expression, insulin resistance and high glucose neutralized this effect.

p53 was found to be a repressor of preadipocyte differentiation [208]. Although its expression was shown to be elevated at the beginning of the process, it was then systematically downregulated [207, 208]. Importantly, p53^−/−^ MEF cells were shown to exhibit lowered insulin-stimulated level of phosphorylated AKT [208]. Other results indicated decreased level of adipogenesis marker genes due to overexpression of p53 [208]. Opposed p53-mediated regulation of white and brown adipogenesis was also revealed [80]. Moreover, p53 knockout mice treated with a high-fat high-sucrose (HFHS) diet exhibited a higher level of fat accumulation in comparison to their wild type counterparts. These results imply that p53 provides a protective effect against diet-induced obesity. On the other hand, p53 was shown to be an indispensable component for proper formation and function of brown adipose tissue (BAT) [80]. In a recent study, p53 was revealed to influence brown adipose tissue via concurrent regulation of body weight and thermogenesis in diet-induced obese mice [209]. It should be noted that p53 was subjected to different types of repression performed on different developmental stages and therefore its manipulation yielded different study results. For example, while mice lacking the p53 gene did not develop diet-induced obesity due to the previously mentioned mechanisms, acute BAT-specific repression of p53 in adult mice resulted in slight weight gain. Interestingly, pharmacologically activated p53 ameliorated body weight by positively influencing thermogenesis in obese adult p53-wild type mice. Notably, brown fat is of great relevance to energy homeostasis, being involved in energy expenditure [210, 211]. While its reduced amount correlates with insulin resistance [212], it is also established as a highly determining factor for metabolic syndrome in mice [213]. In the light of emerging evidence, enhancement of brown fat accumulation constitutes a novel and exciting approach in the battle against obesity [214]. Summing up, p53 can be perceived as a miraculous molecule allowing protection from obesity through confining white fat deposits as well as supporting brown fat formation and functionality. Data obtained by Nakatsuka et al. provide further confirmation for this statement by elucidation of the exact mechanism exerted by the RXR (retinoid X receptor) antagonist HX531 [215]. Its antiobesity effect was accomplished through stimulation of p53/p21 signalling pathway and related to suppressed differentiation of visceral adipocytes. Furthermore, G_0_/G_1_ cell cycle arrest was induced in mature adipocytes, which vastly reduced their hypertrophy. On the contrary, adipose tissue expansion has been attributed to p21 actions exhibited upon HFHS diet [216]. Not only was p21 presumed to have a stimulatory effect towards adipocyte differentiation, but it was also implicated in the promotion of adipose tissue hyperplasia by protecting it from apoptosis. Taken together, p21 was prominently entangled in the creation of obesity and insulin resistance upon high-fat diet treatment [216].

### 5.5. p53 Role in the Formation of Insulin Resistance in Insulin and Noninsulin Target Tissues

Homayounfar et al. evaluated the outcomes of p53 accumulation occurring in peripheral tissues of rats subjected to a high-fat diet [217]. Surprisingly, p53 was found elevated in classic insulin target tissues (adipose, muscle, and liver) and in others (kidney and small intestine). Treatment with pifithrin-*α* (PFT) led to decreased p53 in all examined tissues and proved the crucial role of crosstalk between the ATM/MDM2/p53 and PTEN/Pi3K/AKT pathways. p53-mediated PTEN overexpression was implicated to have a causative impact on the impairment of insulin action. Finally, systemic application of PFT repressed p53 action, simultaneously improving insulin sensitivity, reducing serum glucose level, and enhancing insulin cellular signalling. In the most recent study, obesity-induced p53-mediated deleterious metabolic effects were inhibited by two agonists of *β*-adrenergic receptor in all insulin target tissues [218]. Both of them were shown to stimulate elevation and activation of AKT along with a decrease of p53 and phospho-p53 levels. p53 inhibition was also revealed in noninsulin target tissues. In other words, agonists of *β*-adrenergic receptor cannot be considered as insulin sensitizers as the risk associated with the lessening of p53-dependent antioncogenic cellular protection is too high.

### 5.6. p53 and Age-Related Insulin Resistance

While diabetes is widely known to accelerate the process of aging, the reverse is likewise true. The connection between p53 and age-associated insulin resistance seems to associate with cellular senescence. This issue was firstly raised by Minamino et al. [127], who indicated the causal relationship among obesity, ROS and DNA damage generation, shortening of telomeres, and p53 activation. This sequence of events consequentially led to the introduction of senescent-like changes in adipose depots, thus stimulating an aging-associated insulin resistance. The same was implied in oxidative stress-exposed aging adipocytes [219]. An aging-replicating mouse model, characterized by DNA repair impairment and DNA damage accumulation, indicated the formation of p53-associated cellular senescence in concert with a decline of pancreatic islet mass and the acquirement of a severe diabetic phenotype [169]. Moreover, deletion of ARF-BP1 phosphatase was concurrent with activation of p53 in *β* cells, which underwent age-dependent depletion [168]. Upregulation of p53 in the liver, muscle, and adipose tissue was shown for older and obese wild type mice in comparison to controls [220]. Interestingly, age-related p53 increase was prevented in PTP1B^−/−^ mice, which were proved to be insensitive to diet-induced changes of insulin sensitivity and characterized by a lean phenotype. According to Jeon et al., MCH^−/−^ mice exhibited resistance towards aging-induced accumulation of body mass as well as insulin resistance [221]. In contrast to previous studies, p53 levels decreased in response to aging in spleen and liver of MCH^−/−^ and wild type mice in comparison to their younger controls; however, the magnitude of p53 decrease was lower in MCH^−/−^ mice.

## 6. p53 Polymorphisms in Diabetes and Complications

Besides expression-based findings, the connection between diabetes and p53 does have an underlying genetic basis. Namely, the SNP occurring at p53 codon 72 is constantly examined in the context of susceptibility to diabetes. While subjects with C nucleotide possess a proline variant of p53 (R72), those carrying the G allele have an arginine variant (P72). Genetic studies revealed the p53 codon 72 (Arg72Pro) polymorphism as a predisposing factor for T2D in Finnish, Chinese Han, and European populations [222–224], which highlighted the independence of this variant in the race-specific context. The severity of insulin resistance observed in T2D patients was demonstrated to associate with this polymorphism [225]. Arg72Pro was also connected with waist circumference and determined the association between T2D and BMI [226, 227]. In the most recent study, the above-mentioned SNP was examined in a mouse model and proved to increase the risk of obesity and general metabolic disturbances associated with insulin resistance and T2D [228]. Moreover, carriers of the G allele demonstrated accelerated progression of diabetic nephropathy [229]. A correlation between the Arg72Pro polymorphism and an early age of onset (<6 years) was also found in Italian female T1D patients as well as being associated with the development of polyneuropathy in Russian population [230, 231]. A p53 polymorphism originating from intron 10, A17708T, was reported to coincide with uremic complications in T2D subjects [232]. Interestingly, an association between p53 and diabetes-related complications was shown for diabetic cardiomyopathy [203–205], retinopathy [233], neuropathy [234], nephropathy [235], vasculopathy [183], peripheral arterial disease (PAD) [236], defective wound healing [237, 238], testicular dysfunction [239, 240], thrombocytic complications [241], or augmented apoptosis-based response towards ischemia [242]. Worthy of note, polymorphisms of the previously mentioned p53 transcriptional targets, TCFL2 (rs7903146) and p53INP1 (rs896854), were indicated to underlie the risk for diabetes occurrence [243, 244].

## 7. Conclusions

All of the above findings provide a profound insight into p53 actions triggered by adiposity or expressed upon diabetic conditions. As can be seen, some experimental results are inconsistent, predominantly in adipose tissue. The same applies to p53's role in glucose homeostasis, highlighting both its neutral and highly determining character. Its impressive impact has been shown in other classic insulin-responsive tissues. Clearly, p53 is responsible for the formation of tissue-specific insulin resistance as well as being entangled into the phenomenon of endothelial “metabolic memory” and many diabetic complications. In the majority of studies, p53 was found to be elevated upon increased levels of free fatty acids as well as high glucose stimulus. As both triggers lead to diet-induced obesity, activation of p53 affects numerous cellular phenomena, especially shown in the form of adipose tissue inflammation. Herein, it is impossible to undervalue the link between p53 and NF*κ*B, whose reciprocal interactions were earlier claimed to be predominantly underlain by functional antagonism. The precise nature of their relationship seems to be highly context-dependent, as ROS-stimulated p53 elicits inflammation through an NF*κ*B-dependent mechanism. Herein, disturbed levels of both pro- and anti-inflammatory cytokines along with activated p53 contribute to the exacerbation of glucose intolerance, insulin resistance, and the elevation of serum glucose level. In other words, p53 can affect the entire organism, leading to systemic disturbances, which was brilliantly visualized by pifithrin-*α* administration. Consequently, emphasis should be put on the existence of intricate and mutual regulatory events between p53 and insulin signalling molecules. Simultaneous regulation of AKT and p53 unambiguously forms a core for p53-induced abnormalities. Detailed mechanistic implications of this particular interaction depend on the phosphorylation status of specific sites located within structures of ATM, p53, MDM2, and AKT. The relationship between ATM and p53 seems to be especially intriguing, as obesity-associated DNA damage was found to promote p53 activity. PTEN's contribution to insulin resistance has also been reported before [245], enriching the entire pathomechanism by a strict relationship with p53. PTEN is suggested to be the key player of AKT-p53 crosstalk, taking responsibility for some of the effects induced primarily by p53.

Focusing further on adipose tissue, it seems that p53 influences its fate under physiological and pathophysiological conditions. While it is indispensable for the functionality of brown adipose tissue, it has been revealed to suppress hypertrophy and differentiation of white adipocytes. Prominently, p53 induces senescent-like changes of adipocytes, which can be perceived as both a deepening of the inflammatory state [210] and protection against the deleterious effects of hyperinsulinemia. This becomes especially important in view of the mitogenic and anabolic nature of insulin activities, which are thoroughly different from the cellular effects exhibited by p53. Thus, it constitutes a great challenge for each researcher aiming to therapeutically inhibit p53, as its total deletion might result in the development of a cancerous phenotype. Nevertheless, administration of pifithrin-*α* has been shown to protect from weight gain even upon high-fat diet treatment.

Considering the abundance of p53 activities, its participation in pancreatic *β* cell apoptosis should not be perceived as extremely unpredictable. However, p53 is assumed to strengthen *β* cell mass decrease by participation in the mitochondrial-dependent apoptotic event with simultaneous avoidance of MDM2-mediated degradation. Apparently, p53 takes advantage of its all functional capabilities in the regulation of glucose metabolism and insulin sensitivity.

Elucidation of p53-mediated metabolic regulation is nowadays a highly developing area of research, exposing its impact on an increasing number of prominent biochemical processes. All metabolic activities promoted by p53 are directed towards suppression of oncogenesis as well as neutralization of effects exhibited by insulin. Therefore, insulin resistance seems to be an obvious consequence of the raised level of p53 in obesogenic conditions. However, age-related activity of p53 adds a new dimension to the insulin resistance issue.

While the majority of observations emphasize that p53 is ubiquitously involved in the pathophysiology of insulin resistance, some study results suggest that p53 protects from obesity and diabetes. Apparently, its role in obesity-associated disorders has been undervalued for too long, now pointing to a strong need for further research. Hopefully, it might result in the creation of new and extremely promising therapeutic approaches. However, it is interesting whether p53-mediated regulation of genes responsible for the metabolic regulation described in this review is similar in diabetes-like conditions. Moreover, interactions between p53 and microRNA networks merit further investigation in diabetes-oriented research, as they may constitute a novel idea for the development of therapeutics. The abundance of p53 mediators and effectors do need a profound examination in order to elucidate further regulatory mechanisms controlling p53-associated actions in both tissue-specific and systemic insulin resistance. Certainly, the profound epigenetic research of p53 and the components of its network would be of crucial importance.

## Figures and Tables

**Figure 1:**
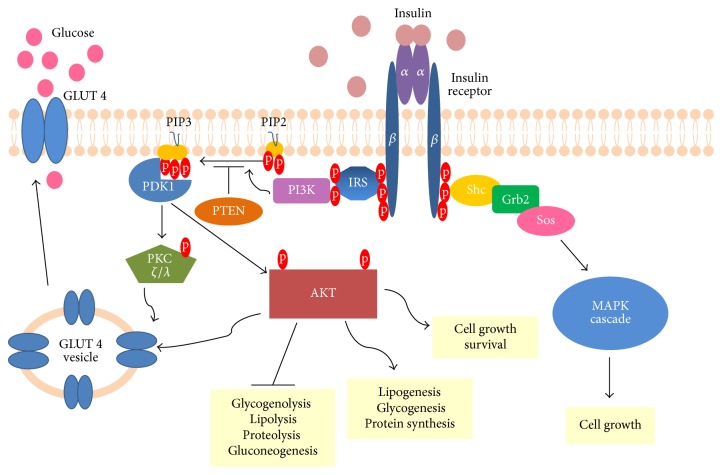
Insulin signalling pathway. Upon insulin stimulation, activation of insulin receptor and thus formation of many phosphotyrosine residues occur. Binding of IRS proteins and their phosphorylation precedes recruitment of PI3K, as well as subsequent PIP2 phosphorylation. Note that PIP3 can be converted to PIP2 through PTEN activity. PDK1 gets activated upon binding to PIP3, followed by the phosphorylation of PKC *ζ*/*λ* and AKT, which stimulate GLUT4 translocation towards the cell membrane and therefore glucose influx. Phosphorylated AKT mediates the repression of glycogenolysis, lipolysis, proteolysis, and gluconeogenesis as well as the activation of lipogenesis, glycogenesis, protein synthesis, and cell survival. The stimulation of insulin receptor is also accompanied by binding of adaptor proteins, known as Shc. The subsequent binding of Grb2 and Sos proteins initiates the MAPK cascade pathway. The activation of AKT and MAPK cascade results in the promotion of cell growth.

**Figure 2:**
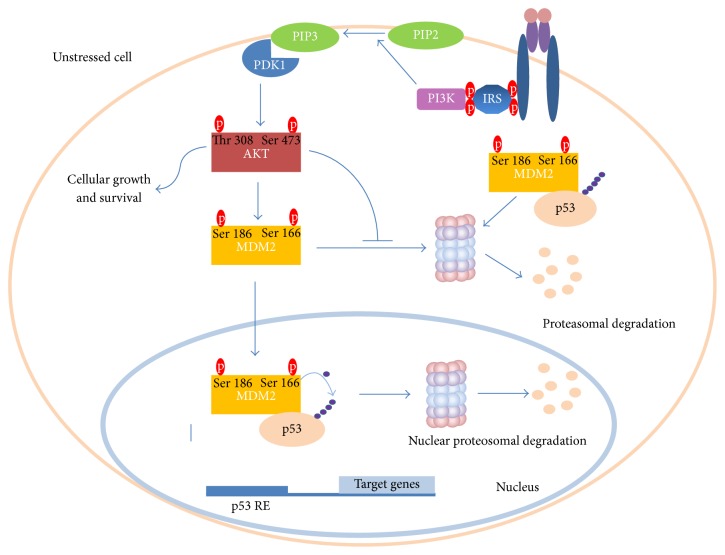
The crosstalk between MDM2/p53 and PI3K/AKT pathways under physiological conditions. Insulin signalling triggers cellular growth and survival. Activated AKT performs MDM2 Ser166 and 186 phosphorylation thereby allowing for the degradation of p53 in the cytoplasm and nucleus. Thus, p53 cannot transcriptionally regulate the plethora of target genes, where these enabling cell cycle arrest, senescence, and apoptosis are of crucial importance. Blue lines denote molecular mechanisms present under physiological conditions. RE stands for response element. See the text for more details.

**Figure 3:**
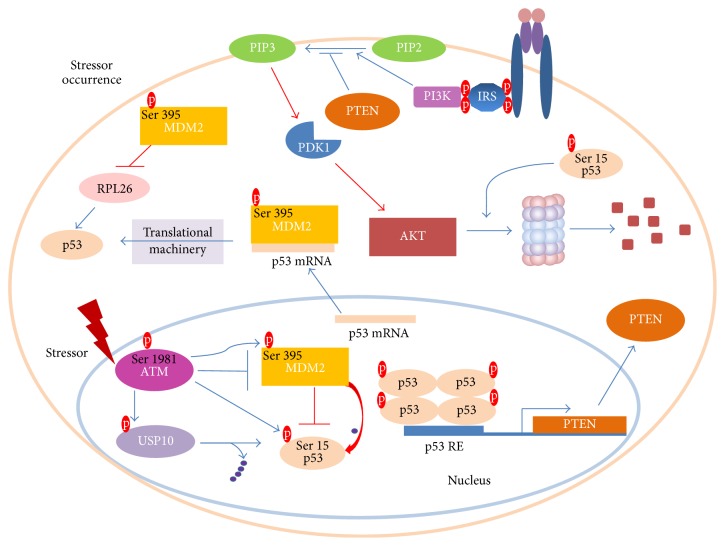
The crosstalk between ATM/MDM2/p53 and PTEN/PI3K/AKT pathways under exposure to genotoxic and metabolic stressors. ATM kinase undergoes activation upon genotoxic stressor occurrence. It conducts MDM2 Ser395 phosphorylation as well as phosphorylating p53 at Ser15. MDM2 cannot negatively regulate p53, which immediately gets stabilized and accumulates. Positive regulation of p53 is performed due to its deubiquitination by activated USP10. Nuclear p53 tetramerization allows for transcriptional regulation of genes involved in provision of antioncogenic cellular protection. PTEN is one of the p53 target genes responsible for the inhibition of insulin signalling. In addition to the blockage of phospho-AKT formation, p53 triggers AKT proteosomal degradation. p53 translation is enhanced by the formation of a p53 mRNA-MDM2 complex and inhibited RPL26 degradation. While blue lines depict phenomena present upon stressor occurrence, red lines represent inhibited signalling events. RE stands for response element. Only a limited number of regulatory events is shown.

**Figure 4:**
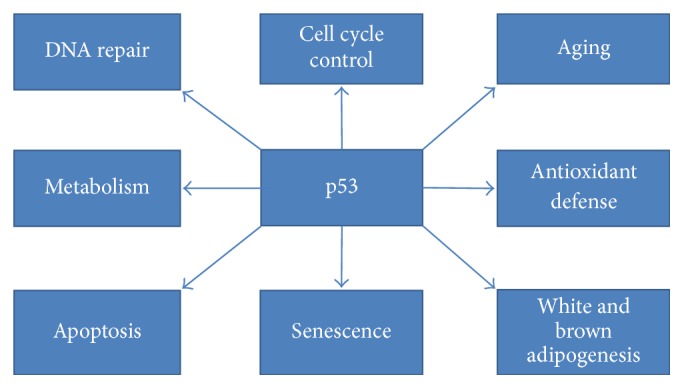
Cellular phenomena vastly influenced by p53.

**Figure 5:**
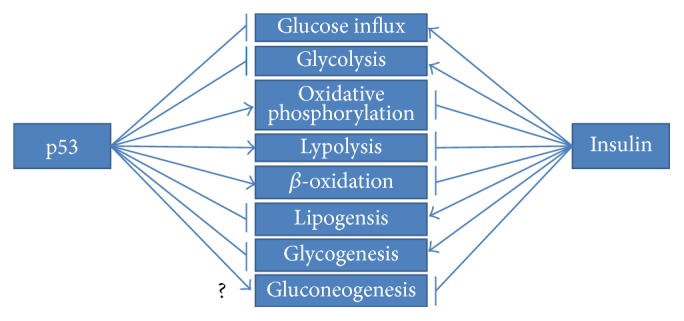
Opposed metabolic regulation executed by p53 and insulin.

**Figure 6:**
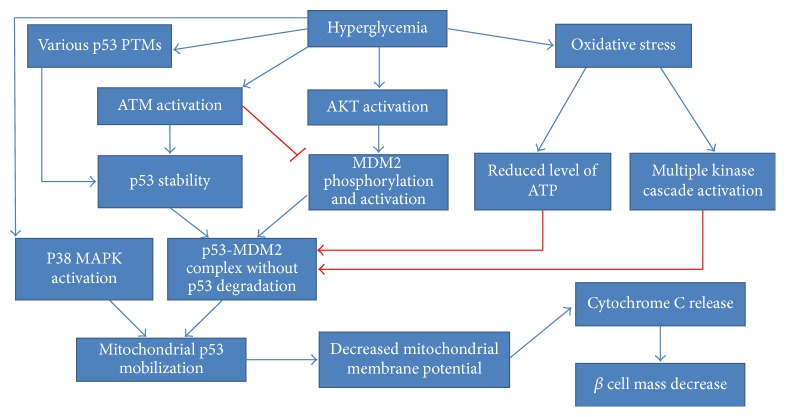
Hyperglycemia-induced changes leading to p53-mediated *β* cell mass decrease. Red lines and arrows denote plausible mechanisms allowing p53 to avoid degradation in spite of p53-MDM2 complex formation.

**Figure 7:**
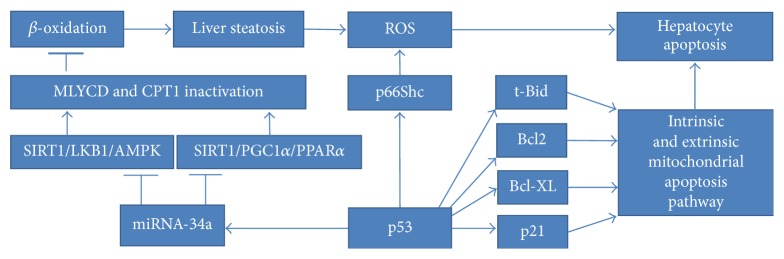
p53-induced cellular events promoting hepatocyte apoptosis.

**Figure 8:**
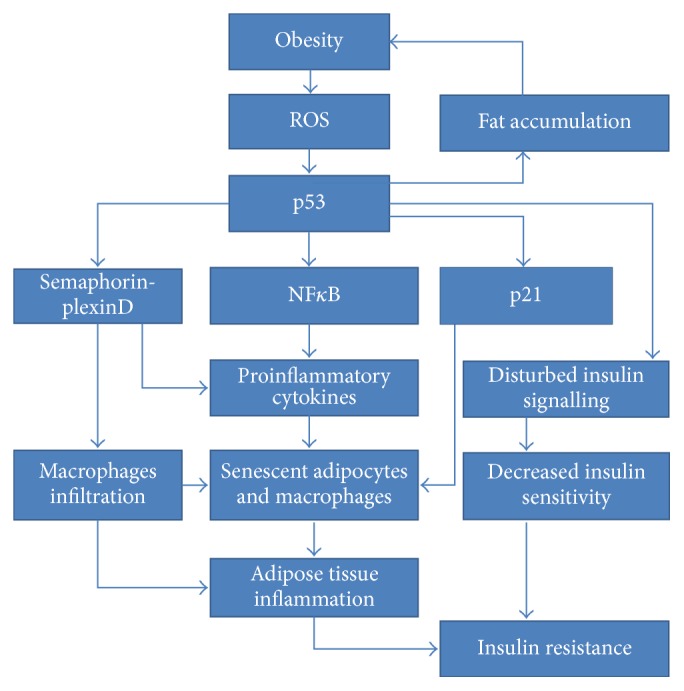
Obesity-induced p53-dependent molecular mechanisms occurring in adipose tissue and leading to insulin resistance.
